# Supplementary material for: Seasonal Trophic Ecology and Diet Shift in the Common Sole Solea solea in the Central Adriatic Sea
Source: Animals (Basel). 2022 Nov 30;12(23):3369. doi: 10.3390/ani12233369 (PMC9736202; doi:10.3390/ani12233369)
Supplement: Supplementary file 1 [file animals-12-03369-s001.zip › animals-1996475-Supplementary.pdf]

Article

Seasonal Trophic Ecology and Diet Shift in the Common Sole *Solea solea* in the Central Adriatic Sea

Emanuela Fanelli [1,2,*], Elena Principato [1], Eleonora Monfardini [1], Zaira Da Ros [1,*], Giuseppe Scarcella [3], Alberto Santojanni [3] and Sabrina Colella [3]

[1] Department of Life and Environmental Sciences, Polytechnic University of Marche, Via Brecce Bianche, 60131 Ancona, Italy
[2] Stazione Zoologica di Napoli Anton Dohrn, Villa Comunale, 80100 Naples, Italy
[3] National Research Council-Institute of Marine Biological Resources and Biotechnologies (CNR-IRBIM), Largo Fiera della Pesca 2, 60125 Ancona, Italy
* Correspondence: e.fanelli@univpm.it (E.F.); z.daros@pm.univpm.it (Z.D.R.)

Supplementary Table S1. Values of %GSI, %HSI, stomach fullness (%), δ^{15}N (‰), δ^{13}C (‰), N%, C% and C/N measured seasonally in n=68 specimens of *Solea solea* captured in the Central Adriatic Sea between January and December 2019 for studying their diets.

Season	Size		%GSI	%HSI	Stomach fullness (%)	δ^{15}N (‰)	δ^{13}C (‰)	N%	C%	C/N
**Spring**	M	mean	0.39	0.86	0.12	11.3	-18.4	19.41	45.87	2.39
		s.d.	0.24	0.23		0.8	0.5	2.77	2.09	0.25
	L	mean	0.71	1.23	0.17	11.2	-17.3	19.93	44.29	2.24
		s.d.	0.15	0.06		0.2	0.5	1.93	1.93	0.28
**Summer**	M	mean	0.38	0.85	0.14	11.0	-17.7	20.57	45.67	2.23
		s.d.	0.31	0.23		0.6	0.5	1.64	1.14	0.15
	L	mean	0.72	1.30	0.29	11.4	-17.4	20.49	45.21	2.22
		s.d.	0.37	0.44		0.6	0.5	1.62	2.45	0.16
**Autumn**	M	mean	0.54	0.93	0.19	11.2	-17.8	20.76	46.02	2.23
		s.d.	0.55	0.20		0.6	0.4	4.23	8.33	0.18
	L	mean	1.41	1.24	0.16	11.4	-17.4	23.82	52.52	2.23
		s.d.	1.06	0.32		0.3	0.2	6.76	13.11	0.25
**Winter**	M	mean	2.69	1.21	0.25	11.9	-17.7	21.28	46.68	2.20
		s.d.	3.57	0.50		0.5	0.5	1.74	1.84	0.15
	L	mean	5.29	1.52	0.25	12.0	-17.7	21.74	45.78	2.12
		s.d.	3.12	0.47		0.6	0.8	2.28	1.57	0.19

Supplementary Table S2. Results of univariate PERMANOVA main and pairwise tests carried out for the factors "Season" and "Size" on the %GSI, %HSI and stomach fullness values measured in n=477 specimens of *S. solea* captured between January and December 2019 in the Central Adriatic Sea for studying their diets. Pairwise comparisons are conducted on the interaction term "Season×Size" for pairs of level of factor "Season". Only comparisons between pairs of consecutive seasons are showed. Level 'M' of factor 'Size'= medium size; Level 'L' of factor 'Size'=large size; df=degrees of freedom; MS= mean square; Pseudo-F= statistic F; t=statistic t for pairwise comparisons; Unique perms= number of permutations; p(MC)= probability level after Monte Carlo test; *=$p \le 0.05$; **=$p \le 0.01$; ***=$p \le 0.001$; n.s. = not significant.

%GSI

Main test for %GSI

Source	df	MS	Pseudo-F	P(MC)
Season	3	37.83	11.47	***
Size	1	28.59	8.67	**
Sea×Size	3	6.86	2.08	n.s.
Residuals	303	3.30		
Total	310			

Pair-wise comparison for factor "Sea×Size" within level 'M' of factor 'Size'

Groups	t	Unique perms	p(MC)
Spring vs summer	0.63	9825	n.s.
Summer vs autumn	1.52	9943	n.s.
Autumn vs winter	1.77	9830	n.s.
Winter vs spring	3.24	9831	**

Pair-wise comparison for factor "Sea×Size" within level 'L' of factor 'Size'

Groups	t	Unique perms	p(MC)
Spring vs summer	0.08	9876	n.s.
Summer vs autumn	3.50	9834	***
Autumn vs winter	0.00	9836	n.s.
Winter vs spring	3.31	9840	**

%HSI

Main test for %HSI

Source	df	MS	Pseudo-F	P(MC)
Season	3	0.88	3.29	*
Size	1	4.19	16.60	***
Sea×Size	3	1.38	1.81	n.s.
Residuals	304	76.80		
Total	311	84.49		

Pair-wise comparison for factor "Sea×Size" within level 'M' of factor 'Size'

Groups	t	Unique perms	p(MC)
Spring vs summer	3.95	9838	***
Summer vs autumn	0.03	9832	n.s.
Autumn vs winter	3.94	9827	***
Winter vs spring	1.66	9831	n.s.

Pair-wise comparison for factor "Sea×Size" within level 'L' of factor 'Size'

Groups	t	Unique perms	p(MC)
Spring vs summer	0.64	9844	n.s.

Summer vs autumn	1.68	9840	n.s.
Autumn vs winter	0.13	9926	n.s.
Winter vs spring	0.78	9916	n.s.

Stomach fullness

Main test for stomach fullness

**Source**	**df**	**MS**	**Pseudo-F**	**P(MC)**
Season	3	0.13	0.58	n.s.
Size	1	0.01	0.41	n.s.
Sea×Size	3	0.22	0.96	n.s.
Residuals	302	0.23		
Total	309			

Pair-wise comparison for factor "Sea×Size" within level 'M' of factor 'Size'

**Groups**	**t**	**Unique perms**	**p(MC)**
Spring vs summer	1.30	9841	n.s.
Summer vs autumn	1.47	9827	n.s.
Autumn vs winter	0.15	9883	n.s.
Winter vs spring	1.93	9894	n.s.

Pair-wise comparison for factor "Sea×Size" within level 'L' of factor 'Size'

**Groups**	**t**	**Unique perms**	**p(MC)**
Spring vs summer	0.21	9840	n.s.
Summer vs autumn	3.05	9837	**
Autumn vs winter	1.16	9950	n.s.
Winter vs spring	0.14	9933	n.s.

Supplementary Table S3. Results of univariate PERMANOVA main and pairwise tests for the factors "Season" and "Size" carried out on the stomach fullness of female and male specimens of *Solea solea* captured between January and December 2019 in the Central Adriatic Sea for studying their diets. df=degrees of freedom; MS= mean square; Pseudo-F= statistic F; p(MC)= probability level after Monte Carlo test; n.s. = not significant.

**Stomach fullness**									
Main test for stomach fullness of FEMALES					Main test for stomach fullness of MALES				
**Source**	**df**	**MS**	**Pseudo-F**	**P(MC)**	**Source**	**df**	**MS**	**Pseudo-F**	**P(MC)**
Season	3	0.19	0.84	n.s.	Season	3	0.06	1.19	n.s.
Size	1	0.12	0.51	n.s.	Size	1	0.07	0.15	n.s.
Sea×Size	3	0.11	0.48	n.s.	Sea×Size	2	0.06	1.26	n.s.
Residuals	300	0.23			Residuals	159	0.05		
Total	307				Total	165			

Supplementary Table S4. List of the %W of the taxa found seasonally in the stomach contents of specimens of *Solea solea* captured between January and December 2019 in the Central Adriatic Sea for studying their diets.

	Season			
	Spring	Summer	Autumn	Winter
**Foraminifera**				
Elphidium	0.04	0.00	0.00	0.21
Unid. Foraminifera	0.00	0.00	0.00	0.40
**Cnidaria**				
Hydrozoa	0.00	0.00	0.00	0.07
**Platyelminthes**				
Cestoda	15.36	5.26	9.64	5.52
**Mollusca**				
**Scaphopoda**				
Antalis inaequicostata	0.00	0.00	0.00	1.57
**Gastropoda**				
Fusinus rostratus	0.00	0.00	0.00	0.13
Oxynoe olivacea	0.67	0.00	0.00	0.00
Turritellinella tricarinata	0.07	2.85	0.32	0.00
Alvania sp.	0.00	0.00	1.99	0.00
Unid. Gastropoda	0.00	0.16	0.00	0.00
**Bivalvia**				
Peronidia albicans	0.00	0.19	0.00	0.00
Petricola pholadiformis	0.00	0.00	0.00	0.46
Corbulidae	0.67	0.00	0.00	0.00
Pectinidae	0.22	0.47	0.00	0.00
Veneridae	2.67	0.00	1.07	3.13
**Unid. Mollusca**	8.23	7.08	18.87	26.84
**Anellida**				
**Polychaeta**				
Lepidasthenia sp.	0.00	0.00	0.00	1.23
Leanira	0.00	0.00	0.00	0.35
Aphroditidae	0.61	0.00	0.00	0.00
Nereididae	0.00	8.26	0.00	0.40
Phyllodocidae	0.00	0.00	0.00	0.17
Glyceriformia	0.00	0.00	0.70	0.00
Sternaspis scutata	0.00	0.00	3.89	0.00
Terebellida	0.00	0.00	0.33	0.00
Maldanidae	0.00	0.00	0.00	0.20
Sabellidae	5.33	0.00	0.00	0.00
Spionidae	0.00	0.00	0.00	0.83
Unid. Polychaeta	22.50	22.00	22.47	10.49

	Season			
	Spring	Summer	Autumn	Winter
**Sipuncula**				
Phascolosoma stephensoni	10.43	0.00	2.14	0.00
Sipunculus nudus	0.00	0.00	0.00	2.12
Sipunculidae	0.00	0.00	1.32	1.54
**Arthropoda**				
**Crustacea**				
**Decapoda**				
Upogebia tipica	0.00	15.21	0.00	0.00
Munida sp.	0.00	0.00	0.00	0.46
Crangonidae	0.00	0.00	0.00	0.14
Caridea	0.00	0.00	1.11	0.00
Unid. Decapoda	0.00	0.00	2.70	0.19
**Amphipoda**				
Leucothoe lilljeborgi	0.00	0.00	0.00	0.26
Ampelisca sp.	0.54	7.41	10.27	5.68
Oedicerotidae	0.00	0.00	0.00	0.14
Gammaridea	0.00	0.00	0.21	0.15
Unid. Amphipoda	1.03	0.00	5.24	0.99
**Stomatopoda**				
Squilla mantis	0.75	0.00	1.03	0.00
**Tanaidacea**				
Apseudopsis latreillii	0.00	3.90	0.00	1.90
Apseudes spinosus	0.00	0.00	0.48	0.41
Unid. Tanaidacea	0.00	1.03	0.00	0.00
**Unid. Crustacea**	0.67	0.50	1.34	1.05
**Echinodermata**				
Paracentrotus lividus	0.00	1.41	0.00	0.00
Holothuria sp.	1.33	0.00	0.00	0.00
Unid. Echinodermata	2.49	2.92	1.51	1.84
**Osteichthyes**				
Fish fragments	0.45	0.00	2.37	1.39
Fish scales	5.41	3.97	3.41	6.52
Larvae	11.64	5.76	0.23	5.43
**Other**				
Shells	6.28	10.36	1.50	12.68
Vegetal rests	0.42	1.26	0.48	0.39
Eggs	0.86	0.00	0.32	2.28
Sand	0.00	0.00	5.06	2.31
Wood	0.00	0.00	0.00	0.18
Unidentified material	1.33	0.00	0.00	0.00

Supplementary Table S5. Results of multivariate PERMANOVA main and pairwise tests carried out for the factors "Season" and "Size" on the results obtained with SCA (%W) carried out on specimens of *Solea solea* captured between January and December 2019 in the Central Adriatic Sea for studying their diets. Pairwise comparisons are conducted on the term "Season" and on the term "Size". Only comparisons between pairs of consecutive seasons are showed. M= medium-size specimens; L= large-size specimens; df=degrees of freedom; MS= mean square; Pseudo-F= statistic F; t=statistic t for pairwise comparisons; Unique perms= number of permutations; p(MC)= probability level after Monte Carlo test; *=p ≤0.05; **=p ≤ 0.01; ***=p ≤ 0.001; n.s. = not significant.

Diet composition (%W)

Main test for diet composition of *S. solea* (%W)

**Source**	**df**	**MS**	**Pseudo-F**	**P(MC)**
Season	3	4545.3	5.70	***
Size	1	5848.5	7.34	***
Sea×Size	3	1299	1.63	n.s.
Residuals	355	797.18		
Total	362			

Pair-wise comparison for factor "Sea×Size" within level 'M of factor 'Size'

**Groups**	**t**	**Unique perms**	**p(MC)**
Spring vs summer	1.91	9949	**
Summer vs autumn	2.01	9942	**
Autumn vs winter	3.00	9951	***
Winter vs spring	2.23	9941	***

Pair-wise comparison for factor "Sea×Size" within level 'L' of factor 'Size'

**Groups**	**t**	**Unique perms**	**p(MC)**
Spring vs summer	0.59	9933	n.s.
Summer vs autumn	1.11	9930	n.s.
Autumn vs winter	2.09	9930	***
Winter vs spring	1.56	9923	*

Pair-wise comparison for factor "Sea×Size" within levels of factor 'Size'

**Groups**	**t**	**Unique perms**	**p(MC)**
M vs L in spring	2.56	9945	***
M vs L in summer	2.25	9944	n.s.
M vs L in autumn	1.14	9955	n.s.
M vs L in winter	0.85	9945	***

Supplementary Table S6. Output of SIMPER analysis conducted on the diet composition (%W) of *Solea solea* within each season both considering overall results and differences in the diets of medium- (M) and large- (L) size specimens. SIMPER analysis was conducted on the Bray-Curtis resemblance matrix of transformed biomass data. Only comparisons between consecutive pairs of seasons are showed. Cut-off for low contribution at 60%. Avg. Ab=average abundance; Avg. Sim=average similarity; Contrib %= percentage of variance explained by the explanatory variables; Cum %= cumulative percentage of variance explained by the explanatory variables.

OVERALL RESULTS OF DIET COMPOSITION

Spring
Average similarity: 21.8 %

Taxon	Av.Abund	Av.Sim	Contrib %	Cum %
Fish larvae	0.21	10.08	46.35	46.35
Polychaeta	0.19	5.36	24.64	71

Summer
Average similarity: 16.5 %

Taxon	Av.Abund	Av.Sim	Contrib %	Cum %
Polychaeta	0.23	6.22	37.64	37.64
Fish larvae	0.12	3.11	18.84	56.48
Shells	0.15	2.75	16.62	73.09

Autumn
Average similarity: 18.3 %

Taxon	Av.Abund	Av.Sim	Contrib %	Cum %
Polychaeta	0.26	12.54	68.56	68.56

Winter
Average similarity: 16.1 %

Taxon	Av.Abund	Av.Sim	Contrib %	Cum %

Spring vs summer
Average dissimilarity = 82.2 %

Taxon	Spring Av.Abund	Summer Av.Abund	Contrib %	Cum %
Polychaeta	0.19	0.23	19.82	19.82
Fish larvae	0.21	0.12	17.72	37.53
Shells	0.1	0.15	13.07	50.61
Fish scales	0.12	0.08	12.73	63.33

Summer vs autumn
Average dissimilarity = 85.6 %

Taxon	Summer Av.Abund	Autumn Av.Abund	Contrib %	Cum %
Polychaeta	0.11	0.26	18.3	18.3
Fish scales	0.18	0.08	13.11	31.41
Ampelisca sp.	0.09	0.13	10.94	42.35
Fish larvae	0.15	0.01	9.81	52.16
Mollusca	0.08	0.07	7.04	59.2
Amphipoda	0.04	0.04	4.93	64.13

Autumn vs winter
Average dissimilarity = 88.9 %

	Autumn	Winter		

Fish scales	0.18	6.84	42.58	42.58
Fish larvae	0.15	4.37	27.17	69.75

Medium size
Average similarity: 19.2 %

Taxon	Av.Abund	Av.Sim	Contrib %	Cum %
Fish larvae	0.18	7.07	36.85	36.85
Polychaeta	0.18	4.69	24.43	61.27

Large size
Average similarity: 11.5 %

Taxon	Av.Abund	Av.Sim	Contrib %	Cum %
Polychaeta	0.2	5.93	51.73	51.73
Fish scales	0.1	2.44	21.25	72.98

Taxon	Av.Abund	Av.Abund	Contrib %	Cum %
Polychaeta	0.11	0.26	18.3	18.3
Fish scales	0.18	0.08	13.11	31.41
Ampelisca sp.	0.09	0.13	10.94	42.35
Fish larvae	0.15	0.01	9.81	52.16
Mollusca	0.08	0.07	7.04	59.2
Amphipoda	0.04	0.04	4.93	64.13

Winter vs spring
Average dissimilarity = 83.4 %

Taxon	Winter Av.Abund	Spring Av.Abund	Contrib %	Cum %
Fish larvae	0.15	0.21	17.15	17.15
Polychaeta	0.11	0.19	15.45	32.59
Fish scales	0.18	0.12	15.13	47.72
Shells	0.05	0.1	8.37	56.09
Ampelisca sp.	0.09	0.02	6.64	62.74

DIET COMPOSITION OF MEDIUM- AND LARGE-SIZE SPECIMENS

M vs L in spring
Average dissimilarity = 89.3 %

Taxon	M Av.Abund	L Av.Abund	Contrib %	Cum %
Polychaeta	0,16	0,31	24,32	24,32
Fish larvae	0,25	0,02	17,47	41,78
Fish scales	0,12	0,14	14,83	56,61
Shells	0,09	0,13	12,01	68,62

M vs L in summer
Average dissimilarity = 85.7 %

Taxon	M Av.Abund	L Av.Abund	Contrib %	Cum %
Polychaeta	0,21	0,26	20,25	20,25
Shells	0,15	0,14	14,14	34,39
Fish larvae	0,16	0,03	12,59	46,98
Ampelisca sp.	0,15	0,07	9,82	56,80
Fish scales	0,10	0,04	9,28	66,08

**M vs L in autumn**<br>Average dissimilarity = 81.1 %				
Taxon	M<br>Av.Abund	L<br>Av.Abund	Contrib %	Cum %
Polychaeta	0,26	0,27	23,49	23,49
Ampelisca sp.	0,15	0,11	14,29	37,78
Fish scales	0,10	0,04	9,98	47,77
Mollusca	0,10	0,03	7,59	55,35
Fish fragments	0,04	0,08	7,11	62,46

**M vs L in winter**<br>Average dissimilarity = 87.9 %				
Taxon	M<br>Av.Abund	L<br>Av.Abund	Contrib %	Cum %
Fish scales	0,20	0,14	14,54	14,54
Fish larvae	0,21	0,02	14,46	29,00
Polychaeta	0,14	0,06	9,44	38,43
Fish eggs	0,03	0,12	9,09	47,52
Ampelisca sp.	0,12	0,04	8,29	55,81
Mollusca	0,07	0,10	7,18	63,00

Supplementary Table S7. Values of seasonal diet diversity observed in n=477 specimens of *Solea solea* captured in the Central Adriatic Sea between January and December 2019 for studying their diets. s.d. = standard deviation.

	Diet diversity (H')	
**Season**	**mean**	**s.d.**
**Spring**	0.45	0.48
**Summer**	0.45	0.55
**Autumn**	0.38	0.42
**Winter**	0.59	0.51

Supplementary Table S8. Results of univariate PERMANOVA main and pairwise tests carried out for the factors "Season" and 'Size" on the δ^{13}C, δ^{15}N and C:N values of n=68 specimens of *Solea solea* captured in the Central Adriatic Sea between January and December 2019 for studying their diets. Pairwise comparisons are conducted on the term "Season" for pairs of level of factor "Season". Only comparisons between pairs of consecutive seasons are showed. M= medium-size specimens; L= large-size specimens; df=degrees of freedom; MS= mean square; Pseudo-F= statistic F; t=statistic t for pairwise comparisons; Unique perms= number of permutations; p(MC)= probability level after Monte Carlo test; *=p ≤ 0.05; **=p ≤ 0.01; ***=p ≤ 0.001; n.s. = not significant.

δ^{15}N and δ^{13}C

Main test for δ^{13}C

**Source**	**df**	**MS**	**Pseudo-F**	**P(MC)**
Season	3	1.68	3.1	*
Size	1	2.68	4.9	*
Sea×Size	3	0.53	1.0	n.s.
Residuals	60	0.55		
Total	67			

Pair-wise comparison within pairs of levels of factor 'Season'

**Groups**	**t**	**Unique perms**	**P(MC)**
Spring vs summer	0.93	9947	n.s.
Summer vs autumn	0.27	9950	n.s.
Autumn vs winter	2.64	9948	**
Winter vs spring	2.02	9954	*

Pair-wise comparison within pairs of levels of factor 'Size'

**Groups**	**t**	**Unique perms**	**P(MC)**
M vs. L	2.21	9954	*

δ^{15}N

Main test for δ^{15}N

**Source**	**df**	**MS**	**Pseudo-F**	**P(MC)**
Season	3	1.47	4.84	*
Size	1	0.17	0.56	n.s.
Sea×Size	3	0.14	0.47	n.s.
Residuals	60	0.30		
Total	67			

Pair-wise comparison within pairs of levels of factor 'Season'

**Groups**	**t**	**Unique perms**	**P(MC)**
Spring vs summer	0.0	9849	n.s.
Summer vs autumn	0.2	9850	n.s.
Autumn vs winter	3.5	9853	**
Winter vs spring	2.7	9840	*

δ^{13}C

Main test for δ^{13}C

**Source**	**df**	**MS**	**Pseudo-F**	**P(MC)**
Season	3.0	0.20	0.8	n.s.

Size	1.0	2.51	1.0	**
Sea×Size	3.0	0.39	1.6	n.s.
Residuals	60.0	0.24		
Total	67.0			
**C:N**				
Main test for C:N				
**Source**	**df**	**MS**	**Pseudo-F**	**P(MC)**
Season	3	0.04	1.1	n.s.
Size	1	0.09	2.7	n.s.
Sea×Size	3	0.01	0.3	n.s.
Residuals	60	0.03		
Total	67			

Supplementary Table S9. Seasonal proportional contribution of each food source to the diet of n=477 specimens of *Solea solea* captured in the Cetral Adriatic Sea between January and December 2019 for studying their diets. SD= standard deviation**.**

	**Proportional contribute**									
**Season**	**Pelagic fish**		**Nephtyidae**		***Ampelisca* sp.**		**Fish larvae**		**Cucumariidae**	
	mean	SD	mean	SD	mean	SD	mean	SD	mean	SD
**Spring**	0.23	0.13	**0.40**	0.11	0.11	0.07	0.09	0.06	0.18	0.07
**Summer**	0.22	0.08	**0.38**	0.09	0.17	0.08	0.08	0.05	0.15	0.06
**Autumn**	**0.33**	0.07	0.28	0.08	0.24	0.07	0.05	0.04	0.10	0.05
**Winter**	0.31	0.12	**0.48**	0.09	0.07	0.04	0.05	0.03	0.10	0.04

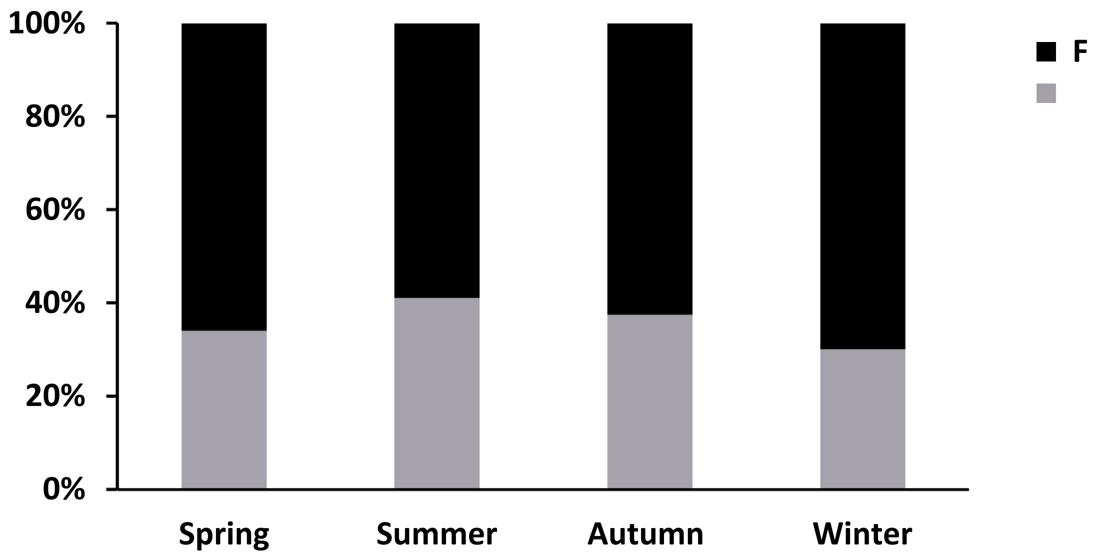

Supplementary Figure S1. Seasonal percentage of female and male specimens of *Solea solea* (n=477) captured in the Central Adriatic Sea between January and December 2019.

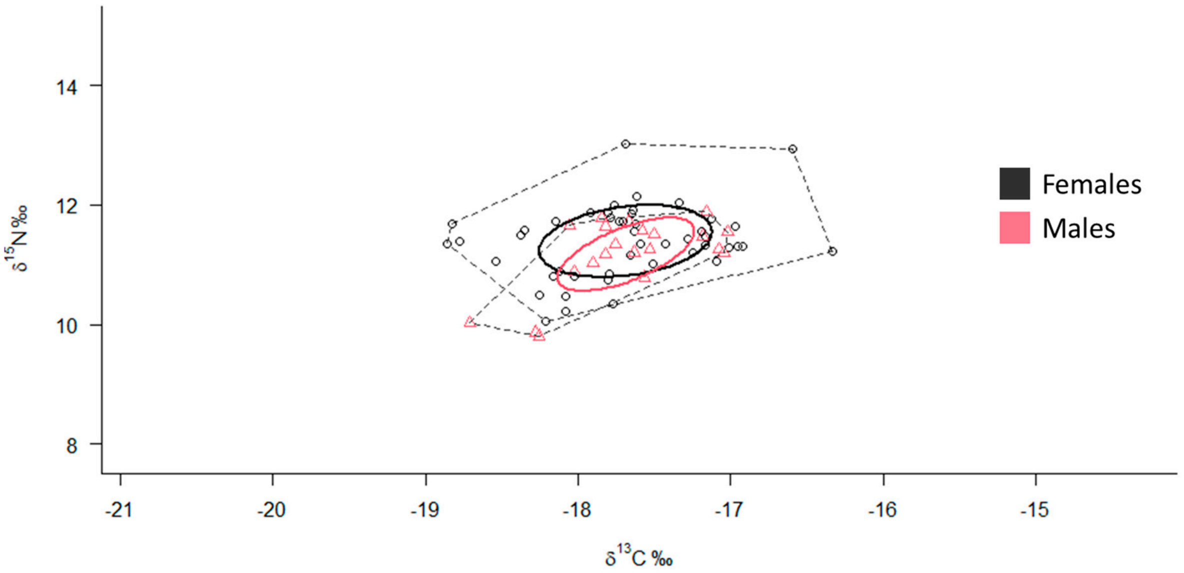

Supplementary Figure S2. $\delta^{13}C$ - $\delta^{15}N$ scatterplot with standard ellipses corrected for small sample size population (SEAC) overlaid for male (n=20) and female (n=48) specimens of *Solea solea* captured in the Central Adriatic Sea between January and December 2019 (p interval=0.4).
